# The Gender Pay Gap: Can Behavioral Economics Provide Useful Insights?

**DOI:** 10.3389/fpsyg.2017.00095

**Published:** 2017-02-02

**Authors:** Renata M. Heilman, Petko Kusev

**Affiliations:** ^1^Department of Psychology, Babeş-Bolyai UniversityCluj-Napoca, Romania; ^2^Department of Psychology, Kingston University LondonLondon, UK

**Keywords:** gender pay gap, behavioral economics, economic games, ultimatum game, gender differences

## A short introduction in behavioral economics

People are faced with numerous decisions every day. Whether we must choose our outfit for the day, which cell phone brand to buy, what college to attend, to buy a car or house insurance, or even when or to whom to get married, decisions are a permanent presence in our daily activities. Behavioral economics is a multi-disciplinary field of study investigating how people make judgments and decisions (Camerer and Loewenstein, 2004; Heilman, 2014). Even though, from a historic point of view, behavioral economics is considered to be a relatively young field of research, the large number of studies that were undertaken and their theoretical and practical implications have made the field of behavioral economics increasingly visible among scholars. More importantly, they have also facilitated contexts to transform behavioral results into social policy programs. Starting in 2010, the UK government launched the Behavioral Insights Team, also known as The Nudge Unit, which was then followed by the Social and Behavioral Sciences Team (SBST), established by the Obama administration in 2014. Both teams aim to apply behavioral sciences, including behavioral economics, in governmental programs in order to increase people's quality of life at lower costs. The efforts of the Nudge Unit and the SBST or other agencies and individual researchers who are trying to improve people's overall quality of life should be supported by the research community through relevant scientific projects and by constantly finding new ways to capitalize research derived knowledge for the general use of a community.

## The ultimatum game

A large proportion of behavioral economics studies rely on various economic games, which have the advantage to depict a decisional situation in a simplified form. The Ultimatum Game (UG, Güth et al., 1982) is a decision-making task that illustrates a negotiation scenario. The standard UG involves two players. The first player, also known as the proposer, has the task of dividing a certain amount of money with a second player, called the responder. The responder can choose to accept or reject the received offer. Should the responder accept the offer, the money is divided between the two players per the proposer's offer. However, if the responder decides to reject the offer, then neither player gets any money. Most frequently, when participating in an UG task, both players are informed regarding the rules of the game, the amount of money that is to be shared and the consequences of their possible actions (Güth and Kocher, 2014). Based on two economic assumptions, namely participants' rationality and their interest in maximizing their gain (Camerer and Fehr, 2006), the normative solution for the UG would be for the proposer to send the minimum possible amount to the responder. For the responder, it would be expected to accept any non-zero amount. Nevertheless, both players behave in a significantly different manner compared to the normative behavior. More specifically, it was found that most proposers offer a larger proportion of the pie to share, approximately 50% of the total amount. Also, responders' behavior deviates from normative expectations because lower offers, of 20% or less of the total amount, are rejected by most participants (Camerer, 2003).

The UG triggers two conflicting reasons that could guide players' behavior. On one hand, normative decisional theories would argue that decision-makers are rational and self-interested, motivated to maximize their gain. Although some people, in certain specific situations, behave rational in the UG, most of the times proposers and responders seem to be guided by some other motive than self-maximization. Judgements of fairness and intentions behind the money allocation decisions are frequently invoked (Loewenstein et al., 1989). There is converging behavioral and neuroimaging data that indicates that people engage in fairness judgements (Brosnan, 2011), due to a concern for reciprocity (Rabin, 1993), or inequity aversion (Fehr and Schmidt, 1999; Tricomi et al., 2010). Therefore, studies suggest people might have an innate sense of fairness that guides their behavior in social interactions and division of a benefit.

Based on the behavioral results obtained playing the UG, the task has established itself as one of the most powerful tools that highlight the limitations of the normative models of decision-making. Since it was first introduced, the UG has been played in hundreds of experimental studies, with numerous methodological variations (Güth and Kocher, 2014).

Many scholars have advanced different theories in their efforts to explain the behavioral pattern in the UG and why the economic normative predictions are violated. Their endeavor opened the possibility to investigate many variables, including methodological modifications, individual differences or even cultural background. A thorough presentation of all these variables is beyond the scope of this paper (for further reading on this topic, see Güth and Kocher, 2014). However, of particular interest for the research community and directly related to this topic are studies that have associated gender differences (Eckel and Grossman, 2001; Solnick, 2001) with decisions related to how much money to send to the responder or when an offer is accepted or rejected.

In spite of the fact that there are many experimental studies related to the UG and how people allocate resources among them, the game's applicability in more ecological environments is less well-investigated. Carpenter et al. (2005) show that there are no significant behavioral differences between UG allocation of college students compared to workers, providing empirical evidence for the external validity of the UG. However, scholars speculate that decision-makers' preferences in the UG might also reflect behavioral differences in real life situations, such as salary negotiations, but direct evidence is missing.

## The gender pay gap

There is an increasing number of studies that show the existence of a gender pay gap, providing systematic proof that, on average, men are paid more than women (Ge et al., 2015; Joshi et al., 2015; Webber and Canché, 2015). It has been estimated that women are paid 23% less compared to their male colleagues, and the pay gap might be even higher for Afro-Americans or Latino minorities (Joshi et al., 2015). Even looking at people pursuing doctoral studies in different domains (Webber and Canché, 2015) or people working in the fast-developing field of IT (Ge et al., 2015) there is a significant salary difference favoring men. Studies show that some of these differences might be due to the fact that women avoid salary negotiations (Eckel et al., 2008; Leibbrandt and List, 2014), or to gender related stereotypes (Reuben et al., 2014; Fabre et al., 2016).

The decisional situation depicted by the UG could be used to test and investigate the factors that contribute to the fact that women are offered less and accept lower salaries than men, while keeping constant the education and professional training levels, total number of working hours during a week or similar job requirements. In a nutshell, the decision to accept a job for a certain salary is similar to the responder's decision in the UG to accept an offer.

## Can behavioral economics provide useful insights and research tools to reduce the gender pay gap?

UG studies have looked at gender differences in offers that are made and accepted/rejected. Most studies indicate that women are offered less compared to men and also that women have higher acceptance rates, including for unfair offers (Solnick and Schweitzer, 1999; Eckel and Grossman, 2001; Solnick, 2001; Eckel et al., 2008). So far, we can only speculate that UG behavior could be related to real life salary decisions. Future studies should take upon the challenge to directly test if there is an association between the two decisional contexts and to what extent UG results could be informative outside the laboratory setting. If systematic research could prove an association between people's behavior in the UG and real life decisional behavior, such as salary negotiation, scholars could connect the two investigative topics with mutual scientific benefits. That is, individual differences that have been associated with decisions in the UG might be investigated if they could also account for the fact that women are offered lower salaries and they usually accept lower payments than men. Various social policies or organizational practices regarding salary allocations for men and women could profit from this scientific cross-fertilization in order to remedy a current discriminating situation.

Until present date, the field of behavioral economics has produced an impressive number of studies regarding our decision-making. Moreover, behavioral economics is already trying to provide useful data that can create or help implement a large variety of social programs designed to increase quality of life. Building on past success, new studies should be designed to further bridge the gap between theory and practice.

## Author contributions

RMH initiated the opinion article and drafted the manuscript; PK provided feedback and suggestions.

### Conflict of interest statement

The authors declare that the research was conducted in the absence of any commercial or financial relationships that could be construed as a potential conflict of interest.

## References

[B1] Brosnan S. F. (2011). An evolutionary perspective on morality. J. Econ. Behav. Organ. 77, 23–30. 10.1016/j.jebo.2010.04.008

[B2] Camerer C. F. (2003). Behavioral Game Theory - Experiments in Strategic Interaction. Princeton, NJ: Princeton University Press.

[B3] Camerer C. F. Fehr E. (2006). When does “economic man” dominate social behavior? Science 311, 47–52. 10.1126/science.1110600 16400140

[B4] Camerer C. F. Loewenstein G. (2004). Behavioral economics: past, present, future, in Advances in Behavioral Economics, eds Camerer C. F. Loewenstein G. Rabin M. (Princeton, NJ: Princeton University Press), 3–51.

[B5] Carpenter J. Burks S. Verhoogen E. (2005). Comparing students to workers: the effects of social framing on behavior in distribution games. Res. Exp. Econ. 10, 261–290. 10.1016/S0193-2306(04)10007-0

[B6] Eckel C. C. Grossman P. J. (2001). Chivalry and solidarity in ultimatum games. Econ. Inq. 39, 171–188. 10.1111/j.1465-7295.2001.tb00059.x

[B7] Eckel C. De Oliveira A. Grossman P. J. (2008). Gender and negotiation in the small: are women (perceived to be) more cooperative than men? Negot. J. 24, 429–445. 10.1111/j.1571-9979.2008.00196.x

[B8] Fabre E. F. Causse M. Pesciarelli F. Cacciari C. (2016). The responders' gender stereotypes modulate the strategic decision-making of proposers playing the ultimatum game. Front. Psychol. 7:12. 10.3389/fpsyg.2016.00012 26834684 PMC4724784

[B9] Fehr E. Schmidt K. M. (1999). A theory of fairness, competition, and cooperation. Q. J. Econ. 114, 817–868. 10.1162/003355399556151

[B10] Ge C. Kankanhalli A. Huang K. W. (2015). Investigating the determinants of starting salary of IT graduates. ACM SIGMIS Database 46, 9–25. 10.1145/2843824.2843826

[B11] Güth W. Kocher M. G. (2014). More than thirty years of ultimatum bargaining experiments: motives, variations, and a survey of the recent literature. J. Econ. Behav. Organ. 108, 396–409. 10.1016/j.jebo.2014.06.006

[B12] Güth W. Schmittberger R. Schwarze B. (1982). An experimental analysis of ultimatum bargaining. J. Econ. Behav. Organ. 75, 367–388. 10.1016/0167-2681(82)90011-7

[B13] Heilman R. M. (2014). Individual Differences in Emotions and Decisions. Implications for Economic Psychology. Cluj-Napoca: ASCR Publishing House.

[B14] Joshi A. Son J. Roh H. (2015). When can women close the gap? A meta-analytic test of sex differences in performance and rewards. Acad. Manage. J. 58, 1516–1554 10.5465/amj.2013.0721

[B15] Leibbrandt A. List J. A. (2014). Do women avoid salary negotiations? Evidence from a large-scale natural field experiment. Manage. Sci. 61, 2016–2024. 10.1287/mnsc.2014.1994

[B16] Loewenstein G. F. Thompson L. Bazerman M. H. (1989). Social utility and decision making in interpersonal contexts. J. Pers. Soc. Psychol. 57, 426–441 10.1037/0022-3514.57.3.426

[B17] Rabin M. (1993). Incorporating fairness into game theory and economics. Am. Econ. Rev. 83, 1281–1302.

[B18] Reuben E. Sapienza P. Zingales L. (2014). How stereotypes impair women's careers in science. Proc. Natl. Acad. Sci. U.S.A. 111, 4403–4408. 10.1073/pnas.1314788111 24616490 PMC3970474

[B19] Solnick S. J. (2001). Gender differences in the ultimatum game. Econ. Inq. 39, 189–200. 10.1111/j.1465-7295.2001.tb00060.x

[B20] Solnick S. Schweitzer M. E. (1999). The influence of physical attractiveness and gender on ultimatum games decisions. Organ. Behav. Hum. Decis. Process. 79, 199–215. 10.1006/obhd.1999.2843 10471361

[B21] Tricomi E. Rangel A. Camerer C. F. O'Doherty J. P. (2010). Neural evidence for inequality-averse social preferences. Nature 463, 1089–1091. 10.1038/nature08785 20182511

[B22] Webber K. L. Canché M. G. (2015). Not equal for all: gender and race differences in salary for doctoral degree recipients. Res. High. Educ. 56, 645–672. 10.1007/s11162-015-9369-8

